# Osteoprotegerin in infection-induced acute inflammatory states in children

**DOI:** 10.1016/j.heliyon.2024.e27565

**Published:** 2024-03-07

**Authors:** Aristeidis Giannakopoulos, Alexandra Efthymiadou, Dimitra Kritikou, Dionisios Chrysis

**Affiliations:** Division of Pediatric Endocrinology, Department of Pediatrics, Medical School of Patras, University Hospital, Rio, Greece

**Keywords:** Osteoprotegerin, Pediatric infections, C-reactive protein, Bacterial infection, Biomarkers

## Abstract

**Background and aims:**

Osteoprotegerin (OPG) is a tumor necrosis factor receptor superfamily member which increases in chronic inflammation and is associated with altered bone turnover and cardiovascular complications. In this study, we investigated whether OPG increases during acute inflammatory states induced by infections in children and correlated its levels with other biomarkers.

**Materials and methods:**

This is a prospective study that included 59 patients with documented bacterial infections, 20 with viral infections and 20 healthy controls. OPG, C-reactive protein (CRP), erythrocyte sedimentation rate (ESR) and white blood cells (WBC) were measured.

**Results:**

OPG serum levels were significantly increased during inflammation induced by a bacterial infection, compared to viral infection and controls (4.17 pmol/l (2.40–12.12) vs 3.2 (1.66–5.33) and 3 pmol/l (2.13–4.76), respectively, p < 0.001). In addition, OPG correlated well with CRP (rho = 0.428, p = 0.0011), ESR (rho = 0.3, p = 0.026), and WBC (rho = 0.266, p = 0.05) only in the group with bacterial infection. The sensitivity of CRP in detecting a bacterial infection was superior to OPG (67.3% vs 38.3%).

**Conclusion:**

This study provides proof of concept that OPG increases differentially in bacterial infections, although with a lower sensitivity than CRP. Further studies are needed to define the role of OPG during the inflammatory states of infection in pediatric infections.

## 1 Introduction

The long-standing research on the field of immunology has revealed a tight interface between bone biology and immunity. This link is expressed through a shared number of certain regulatory molecules such as cytokines, receptors, and transcription factors [1]. More specifically, receptor activator of nuclear factor NF-kB (RANK) and its ligand (RANKL) have an essential role for the differentiation of osteoclasts and the development of immune organs like bone marrow, thymus, lymph nodes and gut associated lymphoid tissue [2]. The third major component of this system is osteoprotegerin (OPG), which is a decoy receptor that blocks RANK signaling by binding to RANKL. OPG, encoded by *Tnfrsf11b* gene, is expressed in the peripheral lymph nodes, osteoblasts and bone marrow stromal cells and acts as an inhibitor of osteoclastogenesis [3]. In addition, it participates in the inflammatory process of chronic diseases, like rheumatoid arthritis, ankylosing spondylitis and Crohn's disease [4,5]. The cause of increased serum OPG levels in these cases, is most likely the activation of pro-inflammatory proteins, such as tumor-necrosis factor α (TNF-α), which upregulates the expression of OPG. Consistent with the above, numerous studies have associated the serum levels of OPG with increased cardiovascular risk [[6], [7], [8], [9], [10]]. Furthermore, studies have shown that osteoprotegerin in serum increases significantly with age in healthy females and males [11,12].

Given that OPG production is promoted by pro-inflammatory cytokines in auto-immune conditions, questions arise on whether the RANK/RANKL/OPG system is activated during the short-lived inflammatory states, triggered primarily by common bacterial and viral infections and if this has any implications on bone or cardiovascular system. To the best of our knowledge, the few studies that have investigated serum OPG levels in inflammatory states caused by infection have been performed only in adult patients of ICU with severe bacterial infections and found increased serum OPG levels of [4,13].

Additionally, of clinical interest would be the differential pattern of serum OPG kinetics during a bacterial versus a viral infection. As infectious diseases constitute a major health problem, it has always been of paramount importance the ability to distinct bacterial from viral infections due to the different treatment modality, better control of rapidly developing worldwide resistance to antibiotics [14] and reduced exposure to possible drug adverse effects [15]. In recent decades, apart from the clinical features and ancillary tests, like the erythrocyte sedimentation rate (ESR), diagnostics has been really helped by the use the so-called acute phase reactant proteins, of which the most clinically important has been the C-reactive protein (CRP).

In this study, we evaluated the OPG levels during the state of acute inflammation, present during an acute bacterial or viral infection in children. We also monitored its levels along the course of the acute inflammatory state and compared its gross kinetic profile with that of CRP to estimate whether OPG could be used as discriminating marker for bacterial infections.

## 2 Patients and methods

In this prospective study 99 children were enrolled. All children were hospitalized for reasons such as severe clinical status, very young age, inability to receive oral antibiotic therapy or required frequent follow-up. Fifty-nine children had a documented bacterial infection. Of those children, 37 were diagnosed with urinary tract infection based on positive urine culture (80% *E. coli* and 20% other microbes – *Proteas mirabilis, Klebsiella or Pseudomonas aeruginosa*). In 22 children, other microbial infections like streptococcal tonsilitis (10 with *group A Streptococcus* – positive throat culture), meningococcal meningitis (1 with *Neisseria mengingitis* – positive cerebrospinal fluid culture), septic arthritis (2 with *Staphylococcus aureus* – positive synovial fluid culture), pneumonia (3 with *Streptococcus pneumoniae*), bacteremia (3 with *Staphylococcus aureus or Streptococcus pneumoniae*), or bacterial gastroenteritis (3 with *Salmonella* spp.). In the last 9 children, the infectious agent was detected by positive blood culture. The twenty subjects of the viral infection group had either a positive PCR for a virus or an immunoglobulin test indicative of acute viral infection (positive IgM for Epstein Barr virus or adenovirus) with negative blood or urine cultures. The age and gender matched control group consisted of outpatients that visited our emergency for other reasons and had no acute infection or any other recorded medical chronic condition. The study was approved by the Ethics Committee of the University Hospital of Patras. All children and their parents were informed, and a written consent was obtained before their participation in the study. Quantitative determination of OPG in serum was performed with enzyme immunoassay (Biomedica Medizinprodukte GmbH & Co KG, Austria). Blood samples were left to clot for 30 min, were then centrifuged at 2000×*g* for 10 min and serum stored at −70 °C until assayed. The assay for OPG detects disulfide-linked homodimers of OPG and intra- and inter-assay coefficients of variation were 3.4% and 7.0% respectively with a detection limit of 0.14 pmol/L. CRP was measured by standard rate nephelometry in mg/dl (Beckman). The sample size of this pilot study was calculated using the software G*Power 3.1 [16] by considering mean and standard deviation of OPG levels observed in previous studies [4,8].

The statistical analysis included mainly non-parametric tests to compare the variables among the groups because all markers showed a skewed distribution. Data were presented as median (min – max) and P values < 0.05 were considered as statistically significant. Mann–Whitney test and Kruskal-Wallis's with Dunn's post-hoc test was used for two-variable and multiple comparisons respectively. The overlapping coefficient of the intragroup distribution comparison between OPG and CRP was calculated using the overlap module for Stata software package [17] Sensitivity and specificity was calculated by using the DIAGT module for Stata [18].

## 3 Results

The median with range (min-max) of OPG, CRP, ESR as well as the WBC in the groups of bacterial infections, viral infections, and the control group, respectively, along with the demographics (age and sex) are described in Table 1.

**Table 1:** Descriptive statistics of the infection markers OPG, CRP, ESR and WBC (median and range) according to the etiology of infection.

	Bacterial Infection group	Viral Infection group	Control group
**Sex M/F**	29/30	9/11	10/10
**Age (years)**	0.65 (0.04–9.91)	1.25 (0.09–8)	2.2 (0.1–5.5)
**OPG (pmol/l)**	4.17 (2.40–12.12)	3.2 (1.66–5.33)	3 (2.13–4.76)
**CRP (ng/ml)**	2.9 (0.01–29.2)	0.01 (0.01–2.4)	0.01 (0.01–0.01)
**ESR (mm/hr)**	30 (2–103)	11 (3–96)	13 (8–20)
**WBC (mm**^**3**^**)**	14,800 (6230–43600)	9225 (3900–16000)	9000 (5600–15060)

**Fig. 1:**
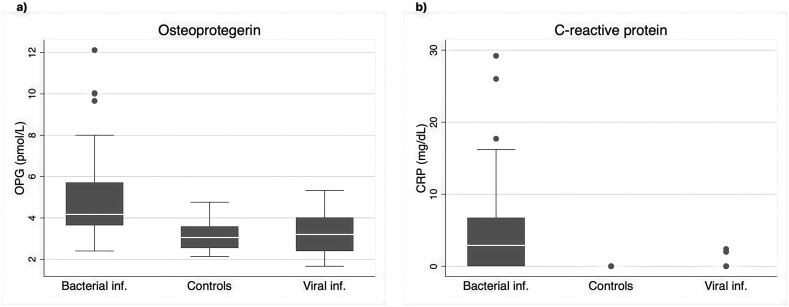
Boxplots of median and range values of OPG (a) and CRP (b) in bacterial, viral infections and controls.

The first point that we observed, was the statistically significant increase of serum OPG levels in children with documented bacterial infections (4.17 pmol/L (2.40–12.12)) compared to the viral infection group (3.2 pmol/L (1.66–5.33)) and controls (3 pmol/L (2.13–4.76)) (p < 0.001). On the contrary, serum OPG levels in viral infections were not significantly increased compared to controls (p = 0.31) (Table 2). Alongside the OPG data, the serum CRP levels, as expected, were significantly increased in bacterial compared to the viral infection group (p < 0.001) (Fig. 1a and b).

**Table 2:** Comparison of OPG and CRP serum levels among bacterial, viral infection and control group using Kruskal-Wallis's with Dunn's post-hoc test with Z-score values and P values reported (asterisks denote P values < 0.05).

Compared groups	N	OPG	CRP
**Bacterial inf. Vs Viral inf.**	59	z = 3.61 P = 0.002*	z = 4.47 P < 0.001*
**Bacterial inf. Vs Control**	20	z = 4.2 P < 0.001*	z = 5.02 P < 0.001*
**Viral inf. Vs Control**	20	z = −0.48 P = 0.31	z = −0.45 P = 0.33

The increase of OPG was tightly correlated to the respective increase in CRP levels only in the bacterial infection group (rho = 0.428, p = 0.0011). Additionally, OPG correlated well with ESR (rho = 0.3, p = 0.026), and WBC (rho = 0.266, p = 0.05) in the same group (Table 3).

**Table 3:** Correlation of different blood indices in bacterial and viral infections denoting Spearman's Rank correlation coefficient (rho) and corresponding P value (asterisk denotes P values < 0.05).

	Bacterial infection			Viral infection		
	OPG	CRP	ESR	OPG	CRP	ESR
CRP	0.428			0.434		
	0.0011*			0.063		
ESR	0.3	0.586		0.168	0.535	
	0.026*	0.0001*		0.4918	0.0181*	
WBC	0.266	0.336	0.227	0.346	0.006	0.306
	0.05*	0.0121*	0.095	0.1458	0.9787	0.2026

**Fig. 2:**
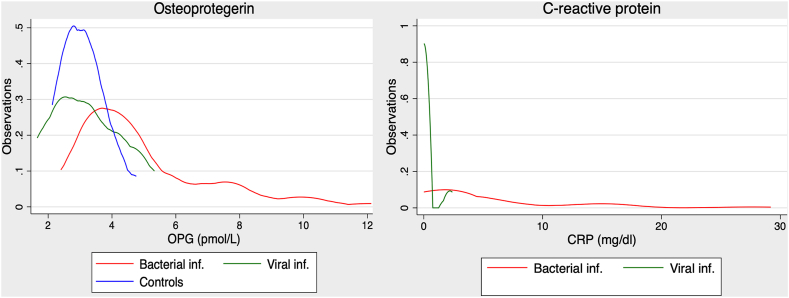
Representation of distribution of observations (Density plots) of OPG in bacterial, viral infections and controls (a) and CRP only in bacterial and viral infections (b).

**Fig. 3:**
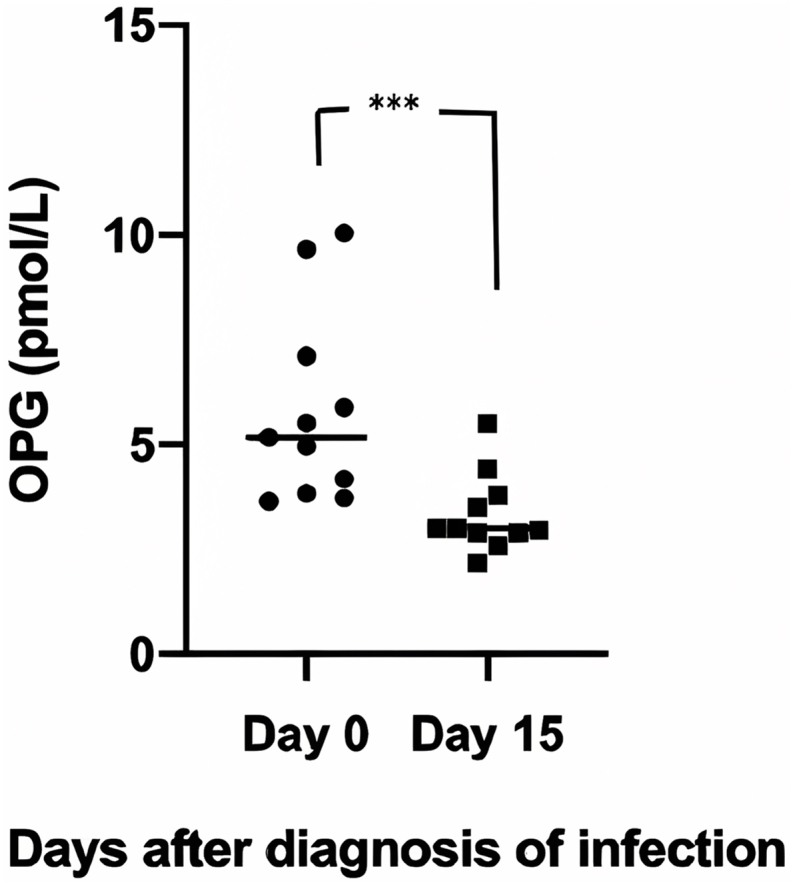
OPG values with medians (lines) at diagnosis and at 15 days after resolution of infection (*** denotes p < 0.001).

Studying the range of values of OPG and CRP in the 2 infection groups (bacterial and viral) and control group, we plotted the densities of OPG, and CRP and we observed, in the case of OPG, a much bigger overlapping values between viral, bacterial and control cases, compared to CRP (Fig. 2a and b). The overlapping coefficient for OPG was 0.31 while for CRP was 0.17 indicating that the overlapping values for serum OPG between the bacterial and viral infection groups was much higher, compared to CRP. In the same context, if we consider the CRP as the reference index for the diagnosis of a bacterial infection, then for CRP value > 0.5 mg/dl, an OPG level greater than 4.82 pmol/L (Confidence intervals: 4.10–5.53) would be considered specific for detecting a bacterial infection. Consequently, the reported specificity of OPG and CRP is equal, as expected, due to the CRP-defined cut-off of OPG. However, the sensitivity of CRP in detecting a bacterial infection is superior to OPG (67.3% vs 38.3%) (Table 4). Finally, in the bacterial infection group, we measured OPG serum levels after recovery (15 days after diagnosis) and found a statistically significant decrease in OPG levels, (5.17 pmol/l (3.64–10.04) vs 3 (2.17–5.5)) (p < 0.005) (Fig. 3).

**Table 4:** Sensitivity and specificity of CRP and OPG for the diagnosis of bacterial infection.

Biomarker	C-Reactive Protein			Osteoprotegerin		
Status	Positive	Negative	Total	Positive	Negative	Total
Bacterial infection	39	20	59	23	37	59
Viral infection	0	20	20	0	20	20
**Sensitivity** (95% CI)	67.3% (52.6%–77.9%)			38.3% (26.1%–51.8%)		
**Specificity** (95% CI)	100.0% (83.2%–100.0%)			100.0% (83.2%–100.0%)

## 4 Discussion

In this prospective study, we investigated serum OPG levels during the transient inflammatory phase induced by a bacterial or viral infection. Secondly, we evaluated the correlation of OPG to the other established biomarkers of infection and its course during and after recovery of the infectious state. We aimed to evaluate the effect of the type of infection, bacterial or viral, on the OPG levels. In this study, we found significantly increased OPG levels only in children with bacterial infection compared to the viral infection group and controls. Most likely, this is caused by the activation of the pro-inflammatory proteins including TNF-α which in turn activates the RANK/RANKL/OPG system as part of the immune reaction to an infectious agent [19]. RANKL is primarily expressed on activated CD4^+^ and CD8^+^ T cells and upregulated by T-cell receptor stimulation during infection [20]. Its receptor, RANK, is highly expressed on dendritic cells and its interaction with RANKL increases their survival and enhances induction of T-cell responses [21]. In this immune response, OPG rather acts as a brake. This is supported by data showing that TNF-related apoptosis-inducing ligand is actively inhibited by OPG [22] and that OPG-deficient mice demonstrate a twofold to fivefold greater capacity to stimulate T-cell proliferation [23] In conclusion, OPG serum levels do increase during acute infection, and this seems to be due to upregulation of expression of all the components of the RANK/RANKL/OPG system. In our study, serum OPG was significantly correlated to all three known indices of infection (CRP, ESR, and WBC) with the strongest correlation to CRP. Interestingly, no substantial change in OPG levels occurred in patients with a viral infection and controls. Whereas in both, bacterial and viral infections, there is an inherent activation of the immune system, it is not known why OPG increases specifically in bacterial infections. Maybe the severity or the nature of viral infection is a factor that determines the degree of immune activation and subsequently the increase of OPG. However, previous studies have found higher OPG levels in HIV infected patients with presumed participation in bone and endovascular damage in this group [24,25]. Interestingly, we did not find increased OPG levels in common viral infections compared to controls. A reason for this may be that HIV is a chronic infection and has a specific imprint on the immune system leading to the progressive loss of CD4^+^ T cells [26].

Focusing on the group with a bacterial infection, comparison between OPG and CRP showed that CRP was more specifically increased compared to OPG. Specificity for OPG is reported to be the same as CRP since its cut off value was defined from CRP's lower positive value. CRP is a marker of inflammation that increases during bacterial infection in response to cytokines IL-1 and IL-6 and has a stable decay rate [27]. Even though bacterial infections are clearly associated with higher concentrations of CRP, its utility for the detection of microbial etiology has been proved to be limited by poor specificity when lower cutoffs are used and on the other side by low sensitivity when using higher cutoffs [28]. Later, other serum biomarkers were introduced, like procalcitonin, that showed a better discrimination power for bacterial infections [29]. The common denominator of the above acute phase reactant proteins is their production in the liver [30]. We also evaluated the gross kinetic profile of OPG levels in bacterial infections and showed that OPG decreases after resolution of infection.

Although it is well established that the activation of the RANK/RANKL/OPG system affects bone metabolism, endothelial function, and therefore vascular health, it is not known whether the short-term effect of bacterial infections on OPG serum levels may have an impact on them. Multiple bacterial infections during the lifetime of an individual may have an accumulative effect. Nevertheless, more studies are needed to evaluate indices of bone metabolism and endothelial function during the transient inflammatory state and its long-term effects.

The limitations of our study were the small number of subjects and the lack of measurement of the TNF-a levels for a better characterization of pro-inflammation status. Further evaluation of the utility of OPG as a biomarker of inflammation of bacterial origin would require a much larger sample of subjects, detailed study of the OPG kinetic profile as well as comparison tests to more selective bacterial induced biomarkers like procalcitonin, given that both are produced at sites other than the liver, which is the production site of CRP.

## 5 Conclusions

In summary, our study, provides the proof of concept for an increase of OPG during acute inflammatory states induced only by bacterial infections and its return to baseline after resolution of the infection.

## Statements

This study was approved (N. 68) by the Ethics Committee of the University Hospital of Patras.

## Data availability statement

Data available on request from the authors.

## CRediT authorship contribution statement

**Aristeidis Giannakopoulos:** Writing – original draft, Methodology, Formal analysis, Data curation. **Alexandra Efthymiadou:** Validation, Data curation. **Dimitra Kritikou:** Methodology, Investigation, Data curation. **Dionisios Chrysis:** Writing – review & editing, Resources, Conceptualization.

## Declaration of competing interest

The authors declare that they have no known competing financial interests or personal relationships that could have appeared to influence the work reported in this paper.
